# Book Reviews

**Published:** 1820-09

**Authors:** 


### CRITICAL ANALYSIS

OF

### RECENT PUBLICATIONS, IN THE DIFFERENT BRANCHES OF MEDICINE AND SURGERY;

### SELECT MEMOIRS, AND HISTORIES OF CASES;

### In the Literature of Foreign Nations.

---

Πατρίδες ἄρα
Ανδράσιν, ὦ πατραῖς ἄνδρες, ἀγαλλόμεθα.

---

### *Cases of Wounds penetrating the Thoracic Cavity; with some Remarks on their Consequences, and on the Application and Effects of Paracentesis of the Thorax for Empyema.* By Baron Larrey.

ALTHOUGH the nature of the species of accident here designated, and the appropriate mode of treatment, had been long since taught and established by Mr. John Bell, we shall adduce an abstract of this memoir, with the confidence that it will present several observations that will be interesting to the surgical practitioner, and calculated to direct the conduct of those not much experienced in wounds of this kind, on many highly important and difficult occasions. If we conjoin with these some statements of a common-place character, the reader will attribute it to our desire to give a perspicuous and connected account of the more important circumstances which ordinarily accompany wounds penetrating the cavity of the thorax. The first principle of the treatment which Baron LARREY originally proposed in France,* is to intercept, as promptly as possible, the access of air to the thoracic cavity, where its presence mechanically compresses the lungs, and embarrasses the respiratory functions, without arresting the hemorrhage furnished by the blood-vessels which have been wounded. By these causes, the blood meeting with an obstacle to its passage through the lungs, accumulates about the heart, and often subsequently in the brain, so as to produce fatal results. Besides this, the lungs are irritated by the air in contact with their external surface, and become inflamed, which state increases the difficulty of respiration, and may itself be immediately the cause of death.

The presence of extravasated blood alone in the cavity of the chest, is not so dangerous a circumstance as those who have not witnessed such accidents are inclined to imagine. When the collection is not considerable, the blood is readily absorbed: it is first isolated by adhesions of the adjacent parts around it, and a cyst is formed, the vessels of which take up the contained blood; or else suppuration of the corresponding part of the integuments of the chest takes place, by which it is evacuated, when the wound is not conveniently placed, or has been healed; or when vent is not given to it by the common operation for empyema. The parietes of the cyst then collapse, and at length approach each other so as to obliterate their cavity.

When the collection is considerable, the natural efforts are ordinarily insufficient to effect its removal, especially if the accumulation has been suddenly produced: the great mechanical pressure of the blood on the vessels of the surrounding pleura prevents their exciting their absorbent faculties; and this membrane often becomes inflamed, and adds to the accumulation by pouring out serous or purulent matter. If a passage for the retained fluid is not opened by art, it at length produces the death of the patient, more certainly when both sides of the thoracic cavity are engaged, by destroying the functions of the lungs, as well as by causing extensive disease of the pleura and other adjacent organs. In this case, then, the operation of paracentesis of the thorax becomes requisite for the preservation of the patient's life. The many instances in which it has been practised with a favourable result, show that it is not in itself a very dangerous operation. The most important circumstances respecting it are to indicate with accuracy, 1°, the cases to which it is appropriate; 2°, the signs of accumulation of fluid

* In the third volume, p. 442, of his *Campagnes Chirurgicales.*

in the thoracic cavity, which the natural efforts are incapable of removing; 3°, the period at which it should be resorted to.

The species of cases to which it is applicable have been already designated, and the inefficacy of the natural efforts to remove the fluid may be generally determined, if nine days have elapsed without this having been either wholly or in great part effected. The signs of an accumulation of fluid in the cavity of the chest, are, a sense of oppression and straitness of respiration; immobility of the lower ribs of the corresponding side, and pain on pressure of the finger in their intervals, through which the finger may distinguish the undulation or impulse of the fluid; difficulty of lying on the opposite side; sound made by the fluctuation of the fluid, often perceptible by the patient himself, and sometimes by the by-standers, especially when the collection is abundant and chronic; the absence of the natural hollow sound on percussion of the chest; and, lastly, it is particularly characterized by an ecchymosis or œdematous tumefaction, situate behind the hypochondre on the side affected. This sign has been invariably present in all the cases seen by the author, and is the only one which is invariably pathognomonic.

Supposing that the necessity of the operation is recognized, it is necessary to determine when it should be performed. It would be imprudent to practise it too early, because this would favour the occurrence of a new internal hemorrhage; and, if the natural efforts were disposed for the absorption of the fluid, this salutary process would be averted. It is proper to wait until the wounded vessels have become healed, consolidated, and the accidents dependant on the pressure and irritation of the accumulated fluid are developed. These ordinarily manifest themselves, so that they may be distinguished from the primary consequences, between the fifth and ninth days. The operation for empyema, for the consequences of wounds penetrating the cavity of the chest, cannot be practised as an efficacious remedy before the seventh day; and is but rarely successful if it is deferred beyond the eleventh, or at the utmost the fifteenth, day. Before the first period we cannot be certain of its necessity; and, if the second be passed over without the evacuation of the fluid, the patient is exposed to imminent danger of perishing from suffocation.

The following abstract of a case detailed by the author, will present many interesting facts to the surgical practitioner, in exemplification of some of the foregoing remarks, as well as show the curious resources of the natural powers for remedying of injuries of the kind under consideration.

An athletic grenadier, twenty-two years of age, received, in a duel, the 7th of September, 1818, at five o'clock in the morning, a thrust from a straight sword, by which he was run completely through the chest, at the upper part of the left side. His adversary had a great deal of difficulty in withdrawing his sword from his body. Notwithstanding the abundant hemorrhage which followed the extraction of the weapon, the soldier did not fall, and he reached, on foot and without the assistance of any person, the nearest house, which was, however, at a considerable distance from the place of combat. There his wounds were bound up with napkins, and he was immediately afterwards carried to the military hospital *Gros-Caillou*, where he was received and attended to by Baron Larrey.

The great quantity of blood which the patient had lost, had produced such a state of debility, that his face and lips were completely pallid, his voice extinct, his eye-lids half closed, and his eyes dull and motionless, his extremities perfectly cold, and his pulse hardly sensible.

On examining the wounds, that by which the sword entered was found to occupy the anterior part of the space between the first and second ribs; it was quite close to the sternum, which was itself a little cut: it was about ten lines in length. The second, made by the point of the sword in passing out of the chest, was situate between the superior and posterior angle of the scapula and the third dorsal vertebra, and corresponded with the space between the second and third ribs: this was only six or seven lines in length. The edges of the wounds and the surrounding parts were considerably tumefied by emphysema. On the edges of the wounds being separated and made to correspond with the openings in the intercostal muscles and pleura, florid and frothy blood escaped from them in powerful jets. These emissions were followed by symptoms of suffocation, and the patient seemed to be menaced with instant death.

The wounds were extended externally, so that no cul-de-sac or pouch might exist under the integuments; and, to bring on a parallel the openings in the integuments and those of the immediate parietes of the chest, they were respectively brought together by adhesive plaster, which, with compresses and bandages, constituted the dressing employed. The application of some cupping-glasses with the scarificator, in their immediate vicinity, removed the surrounding emphysema. The whole of the patient's body was rubbed with hot camphorated oil of chamomile, and the extremities and abdomen afterwards covered with hot flannel. The state of the patient soon improved; his respiration became less laborious and deeper; his pulse and the warmth of the body became gradually developed; he recovered the use of his senses; and in a few hours every thing indicated that the internal hemorrhage had ceased. Some chicken-broth with nitre was ordered to constitute his drink and the whole of his nourishment.

On the evening of the same day some fever, with all the signs of re-action, appeared. Dr. GRIMELLE, a military surgeon, to whom the watching of the patient was particularly confided by Baron Larrey, then bled him, and applied cupping-glasses over the whole of the left side of the chest, some with, and others without, the scarificator. The dressing was not disturbed. The patient remained tolerably calm during the night.

On the ensuing morning, the pulse was rapid and thrilling; the heat of the surface was excessive, the cheeks red; there were deep and acute pains in the region of the wounds, and expectoration of a rather large quantity of dark blood. Phlebotomy was repeated; and iced, acidulated, mucilaginous drinks, to be taken alternately with the chicken-broth, laxative glysters, and nitrated anodyne emulsions during the night, were prescribed.

On the third day and during the night, the edges of the posterior wound were suddenly separated, by an effusion of blackish blood, which recurred on the removal of the dressing. This effusion, far from alleviating the patient, augmented the alarming symptoms; and it was feared that he would die whilst the dressing was being renewed. The anterior wound was dressed at the same time, which had also been forced open; the camphorated embrocations repeated, and the patient placed in a fresh bed.

From this time the patient continued to get better, and he became perfectly tranquil. He remained in this state until the seventh day from that of the accident. A little blackish blood had, however, flowed from the hiatus which had been left in each of the wounds; but they no longer gave access to the external air, and their cicatrization was gradually taking place. In the night of the seventh day, the patient began to suffer an extremely distressing sense of uneasiness, and he could not get an instant of repose. On the eighth day, his pulse was quick and compressed, but there was but little oppression or difficulty of breathing, and the patient did not complain; he suffered but little, and believed himself pretty well. Notwithstanding this, an attentive examination of his general condition and that of his chest, led Baron Larrey to believe that there was a collection of fluid in the left thoracic cavity; and he proposed to perform the operation for empyema the next day, if the characteristic symptoms of such a collection were present in the same degree. As this was the day for Baron Larrey's clinical lecture, he made this patient the subject of it. There was a great concourse of medical men present, many foreigners, who, having examined the patient's chest by percussion, could not believe that any empyema existed; and, notwithstanding the Baron's observations, they disapproved of the operation. But the symptoms of the accumulation, especially the ecchymosis with œdema at the posterior part of the left hypochondre, assured him that he was correct in his opinion; and he proceeded to the operation. It was performed at the lowest and most posterior part of the thoracic cavity of the side affected, with the precaution of not having a direct relation, in the natural state of the parts, between the opening in the integuments and that in the intercostal muscles. After a small incision had been made through the internal layer of intercostal muscles with a scalpel, this incision was enlarged with a probe-pointed bistoury. About five pints of a grumous fluid, of the colour of wine-leys, escaped. A roll of linen, with a thread attached to it, covered with cerate, was passed into the opening in the thoracic paries, to prevent the adhesion of the superior flap of the integuments to the edges of this opening; and the wound was dressed with plasters, compresses of lint, and a bandage, so as to leave vent for any fluid that might be present.

From the instant at which the fluid began to escape, the patient felt some alleviation, which continued to increase till the operation was terminated. The pulse developed itself, and all the functions were restored. The same broth, a little Bordeaux wine, and a pectoral ptisan, were prescribed. He had two or three hours sleep in the ensuing night.

On the following day, a considerable quantity of the same dark fluid was found to have been evacuated: this appeared for several days, with some variation of its colour. On the sixth, it was of a whitish-grey hue; by the ninth it had assumed a purulent appearance, and was inodorous. It then gradually diminished in quantity. The wounds made by the sword cicatrized but slowly, especially the anterior, notwithstanding the care that had been taken to produce their immediate union.

During the first month or two, slight febrile disturbance, with signs of gastric disorder, was occasionally manifested; which was treated with gentle emetics, a cold infusion of cinchona in distilled water, or other bitters and neutral salts, according to circumstances. To these commotions, which were followed by much emaciation, there succeeded several curious pheaomena.

First, the beatings of the heart perceptible externally, which had disappeared in consequence of the displacement which the organ had suffered from left to right from the presence of the accumulated fluid, at first re-appeared a little on the left of the sternum, then gradually extended from it until they were sensible in the natural situation, and successively still more deeply and backwardly, so as not to be evident to the touch at the time the recovery of the patient might be confidently expected.

Secondly, the pulsations of the radial arteries in the two arms presented a sensible difference. Those of the left were well-marked, regular, and uniform; those of the right, precipitate, and interposed with a half-pulsation, with a character of undulation and retrograde locomotion.

Thirdly, the veins of the left arm were not apparent; whilst those of the right remained tumid during the twenty-five or thirty days which immediately followed the operation.

These phenomena continued for a month or six weeks; but at length the pulse in the two arms became in harmony with each other and regular, though the left remained the weakest.

By the fortieth day, the patient felt himself pretty well, and he continued still to improve in health. The pus which escaped from the wound made in the operation had a good appearance, and was reduced to a small quantity. Its evacuation was often accompanied with the issue of membraniform or flocculent slips, which the author attributed to exfoliation of the internal surface of the costal pleura. The wounds made by the sword had entirely cicatrized by the thirty-fifth day.

It could be remarked daily, in a very sensible manner, that the parietes of the left side of the chest gradually fell in and approached each other, so as to reduce the volume of the interior cavity: the left nipple had descended an inch lower than the right; the shoulder of the same side was depressed in the same proposition, and the intercostal spaces were considerably reduced in extent. The right cavity of the chest had enlarged, the author says, in a relative manner.

On the hundredth day after the operation, the patient ate some light food, and walked about the wards of the hospital for several hours in the day, without any support. The wound made in the operation remained fistulous, but it gave vent to but very little pus, which was inodorous and of a good appearance. The patient arrived at the conclusion of the fourth month from the receipt of the wound, and every thing concurred to assure his recovery. But this soldier, naturally of a headstrong, self-willed, and extremely irascible disposition, now became weary of the regiment to which he was submitted, and, determining to resist his inclinations no longer, gave himself up to every excess which he could commit. One amongst these was the use of spirituous liquors, which he clandestinely procured. The consequences were, an attack of violent inflammation of the heart and its envelopments, which terminated in death in forty-eight hours; the patient going off in a paroxysm of rage, uttering every sort of imprecation, on the 11th of January, 1819, on the hundred and twenty-fifth day after the accident.

The body was opened twenty-four hours afterwards, in the presence of Dr. Ribes, and the other medical officers of the hospital.

The capacity of the left side of the chest was found to be reduced to two-thirds of its natural space: the mediastinum had extended its adhesions to the left as far as the line of union of the cartilaginous with the osseous portions of the ribs. The heart and pericardium occupied the greater part of this cavity, and were situate more backwardly and to the left side, than in the natural state. The portion of the lung which had escaped the effects of the compression of the effused fluid was hepatized, and covered with a villous false membrane which adhered to the pericardium, whilst the latter was throughout so intimately united with the surface of the heart, that it was only with difficulty a small portion of that membrane could be separated from it. The diaphragm was considerably vaulted on this side. The ribs were thicker than those of the opposite side, as well as more approached together. The reduction of this thoracic cavity was such, that there remained only a small space of a conical form, at the bottom of which was a little purulent matter of a whitish colour. The parietes of this cavity were lined with layers of membraniform filaments.

The wound made in the operation was fistulous, and situate precisely at the lowest point of the cavity. The wounds made by the sword were perfectly cicatrized.

On examining attentively the course which the weapon had taken in traversing the chest, it was found that the internal mamillary artery had been completely divided; the superior lobe of the left lung completely traversed, but, whether above or below the arch of the aorta, is not stated; and the intercostal artery divided in the situation of the posterior wound.

The manner in which the natural efforts had effected, to a great extent, the obliteration of the cavity left by the partial destruction of the lung, is a very curious circumstance. Until this obliteration was complete, that is until the cavity was only equivalent to the heart and the remaining portion of lung, it must be evident that the wound made in the operation could not heal, or, if it did completely close for a few instants, that it must re-open, unless a permanent effusion of fluid was maintained to fill up the space left by the heart and lung, as a vacuum could not be maintained; and the obliteration of the air-cells, by the hepatized state of the lung, would not permit of the air rushing in by the trachea to fill up the vacancy, by developing the organ just mentioned. This fact is worthy of consideration in cases of hydrothorax consequent on inflammation of one or both lungs, by which these organs have become hepatized to a greater or less extent. As they cannot in this state develop themselves so as to fill the thoracic cavity, it is clear that the water cannot be removed. Every effort to effect its removal by the vessels would tend to create a vacuum, which must in some way or other be obviated. Perhaps these views, with the facts witnessed in the case related by Baron Larrey, should be taken into consideration, in explanation of the contraction of one side of the chest after pleurisy and hydrothorax, described by LAENNEC. The causes he proposes for it have probably some influence in producing the effect under consideration, but they do not seem to be alone adequate for it.

We find that our limits will not permit us to proceed further at present in the consideration of this memoir; but, as the rest of it is made up of several other cases, illustrative, like that already noticed, of the most interesting phenomena attendant on the species of accident under consideration, we shall give a simple transcription of those cases in the next Number of this Journal.

---

***The Natural History of the Medicines, Aliments, and Poisons, taken from the Kingdoms of Nature.*** **By J. J. VIREY, Doctor in Medicine of the Faculty of Paris, Member of several Learned Societies, Professor of Natural History at the Atheneum at Paris, Master in Pharmacy, late Pharmaceutist-in-chief to the Military Hospital of the Val-de-Grace, Associate and Correspondent of several Foreign Academies, &c. 8vo. pp. 570. Remont and Ferra, Paris, 1820.**

**[In continuation from page 168.]**

THE next part of this work is devoted to " the natural history of the odours of aliments and medicines, with their classification, and some observations on their nature and diverse modifications." But little that is satisfactory is effected in the way of classification of either properties or qualities on this subject, although it has been extensively treated on by numerous writers. It is not possible to reduce them to any classes, for they have no very precise analogies, and are almost as various as the diverse bodies in nature. But little has been done even in appropriating appellations to them, so that ideas of them may be conveyed by language.

M. Virey attempts another mode of making something like an arrangement of them. He treats, in succession, of the odours of aliments, of medicines, between which no very precise distinctions can certainly be made; under which heads he classes odours, according to their resemblance with those of several well-known powerfully-odorific substances. He then considers odours in respect to the use made of them for luxurious purposes; and the effects of combination, putrefaction, heat, air, light, and various chemical re-actions on them; and, finally, of their chemical nature.

Powerfully-odorous bodies are generally also strongly sapid; and animals in search of their food examine them previously to resorting to taste. Substances of a disagreeable odour to any particular animal are but rarely fit for the nutrition of that animal. Some substances of a disagreeable smell are, however, pleasant to the taste of an individual, and these are generally proper food for it. M. Virey distinguishes the odours of aliments into the following species: 1, the musty; 2, the oleraceous; 3, the leguminous; 4, the umbelliferous; 5, the antiscorbutic; 6, that of fruits; 7, the dulcid; 8, the oleaginous; 9, that of raw and cooked flesh; 10, that of fish; 11, that of garlic; 12, the aromatic. We pass over the exemplifications of these, as not being of much importance or utility, any more than the classification itself, which is open to many objections.

Several medicines, the author remarks, seem to act on the body only by means of their odour; and many of them, as some of the purgatives, lose their medicinal qualities with their nauseous smell. Thus, when rhubarb is deprived of its smell by being torrefied, it loses its purgative quality, and becomes only astringent.

Some very disagreeable odours seem to produce deleterious effects on the animal body only by their impression on the organ of smell. Some odours have singular effects on the nervous system, and others excite especially certain organs; but we hardly know what degree of credit to attach to the stories related in exemplification of this proposition.

Linnæus formed seven classes of odours of medicines; namely, the aromatic, fragrant, ambrosiac, alliaceous, hircine, fetid, and nauseous. M. Virey proposes a different arrangement, which is very objectionable; especially because the supposed effects of certain plants on the human body are made the basis of several of the classes, and the source of their applications. They are, 1, the nauseous; 2, the vinous or narcotic; 3, the acrid or corrosive; 4, the hircine; 5, a somewhat similar odour, which may be termed aphrodisiac; 6, that of emmenagogues; 7, the nidorous; 8, the carminative; 9, the bituminous; 10, the strong or penetrating; 11, the camphoric; 12, the aromatic; 13, the balsamic; 14, the resinous; 15, the gummoresinous; 16, the muscal or ambrosiac; 17, the citronic; 18, that of the lotus species of plants; 19, the acerb; 20, that of kernels containing prussic acid.

The author has arranged the several medicinal substances under those classes; but our limits oblige us to pass over these particulars to matters of more importance. He has not been so successful in designating the qualities of medicinal substances by their odours as by their colours, though much is certainly determined even by the former means.

The rest of the disquisition on this subject contains many curious and interesting observations; but these are not so closely allied to the especial object of this Journal as to detain our attention on the present occasion. This is also the case with the greater part of that on the tastes of medicines. Besides, the varieties in the mode of sensation of different individuals prevent any very precise conclusions respecting the wholesome or unwholesome properties of plants being formed from their agreeable or unpleasant taste. Though it may be stated in a general manner, that those which are nauseous to the taste are more or less noxious to man in the state of health. Besides the objections to the indications of taste above mentioned, it may be observed, that what is poison for one animal is food for another, even among those whose organization is not considerably at variance. Thus, the *phellandrium aquaticum*, which is poisonous to man and to the horse, is eagerly sought after for food by the bullock.

M. Virey distinguishes the tastes of medicines into excitant, acrid, bitter, aromatic, saline, styptic or astringent, virulent, acid, sweet, insipid, oily, mucilaginous, and narcotic.

The qualities of the simple excitants are sufficiently distinguished by the sense of the appellation. Those which are acrid admit of some subdivisions which merit consideration. The alliaceous acids, the type of which is garlic and other analogous vegetables, as squills, colchicum, the bulbs of the anthericum and amaryllis, and, in general, of several of the lilies, asphodels, the irides, &c. although this state is, in some of these instances, joined with a nauseous, purgative, and even poisonous, principle. These substances strongly excite the system, and especially the mucous membranes. The antiscorbutic acrids, the type of which is the radish, cresses, cochlearia, mustard, and, in general, all the cruciferous plants. This acridness is fugacious, and disappears on desiccation of the vegetables. It is decomposed also by heat, and furnishes either sulphur or sulphurated hydrogen. Plants containing it excite, especially, the secretion of urine. The spicy acrids vary but little in their qualities from simple excitants. The caustic acrids are for the most part dangerous when taken inwardly. Many plants which grow in water become then possessed of this quality in a greater degree than when they grow on dry land, and several of them acquire also proportionate poisonous qualities. Of this kind are the polygonum hydropiper, cicuta and several other of the umbelliferæ, sium, phellandrium, the ranunculacea, the species calea and arum, the nymphœæ, the vernal plants of moist soils, as the anemone pulsatilla, the chrysosplenium, &c. Other acrids, naturally caustic, are the aconites, cevadilla, clematis, coculus indicus, the euphorbia, gratiola, the hellebores, the delphinia, staphysagria, the ranunculacea, savine, toxicodendron: several milky plants, as the apocynum, the thymelœa, &c. inflame the skin, and are poisonous when taken into the intestinal canal. Many of them act as drastics, or as emetics.

"The bitters," says M. Virey, "constitute one of the savours the most unpleasant to the taste, and nature leads us to shun them involuntarily. This is indeed with reason, because the greater part of the most intense bitters are poisonous; others purge; others are joined with nauseous or fetid savours which excite vomiting. There are some of them, however, which have nothing deleterious, nor even very disagreeable, in them: these are the aromatic bitters, and the bitter astringents. The latter are excellent tonics and febrifuges; they strengthen the organic system, excite the appetite and digestion: after a long use they enervate, harden the animal fibre, and produce emaciation. They are also taken as antiaphrodisiacs; they oppose the development of acidity in the intestinal canal, and destroy worms. They are successfully employed against arthritic and hypochondriac disorders; they resist putridity, and solicit the activity of the liver or the secretion of bile."

We can distinguish four species of bitterness: 1. The astringent bitter, the type of which is in the cinchona. The vegetables possessed of this property prevent putrefaction, destroy worms and parasitic animals, oppose more or less the course of intermittent fevers, and give tone and vivid colour to the body. 2. The odorific bitters; such are especially absinthium, chamomile, southernwood, inula, the euphatoria, spikenard, milleflower, scordium, zedoary, cascarilla, serpentary, &c.: these are more irritating and stimulant than the foregoing, and excite more particularly the nervous system. 3. The fetid bitters are, generally, vermifuges and emmenagogues; such are artemesia, gum ammoniac, the aristolochiæ, assafœtida, ballota, galbanum, the hop, rue, valerian, tansy, &c. 4. The nauseating bitters: these are purgatives and sometimes emetics, and they become even poisons in very large doses. Thus aloes, colocynth, elaterium, bryony, nux vomica, and several of the agarics, purge violently. The cusparia is included amongst the foregoing by the author, but improperly, for it is not properly a purgative. Several of the bitter lichens, the species of false cinchona having the stamens longer than the tube of the corolla, of persoon, are emetic. It appears that the purgative principle is particularly allied with nauseating qualities, since, on gently torrefying rhubarb and scammony, or long boiling of asarum, senna, bryony, ricinus, &c. these purgative properties are dissipated with their nauseous taste.

The principle of the action of the aromatics appears to reside especially in volatile oils or benzoic acid, or in balsams and resins more or less soluble in our fluids. They affect particularly the nervous system, almost as simple excitants.

The virulent savours are distinguished by the author into those of the mineral, and those of the organic kingdom. Substances possessing this quality are more or less deleterious to our system. The most virulent savours of the vegetable kingdom are those of several champignons, of the thymelœa, the plumbago, the greater part of the acrid and bitter apocynea, as the verberæ, the strychnos, the milk of the asclepias and cynanchum; that of the lobeliæ tupa and urens is caustic; the bulbs of several ranunculacea, as the sceleratus, flammula, and thora; the aconites, the clematis flammula; some of the species menispermum, such as the coculus and the rhus toxicodendron; the acrid milky juices of the euphorbiaceæ, especially of the hippomane, sapium, adelia venenata, that of the ficus toxicaria and septica, of the ipo toxicaria, &c. are very poisonous, and of an insupportable taste. The simple sweet and almost insipid, (*fade*,) as well as mucilaginous savours, are devoid of deleterious qualities. Some mucilages have nauseous or fetid qualities joined with them, as in the narcissus, the asphodels, the irides, and gladiolæ, which are poisonous.

The narcotics may be distinguished into, 1, the inebriating; 2, the nauseous.

The inebriating narcotics are alcohol, fermenting liquors: the hop, the arniac, eupatorium, colium, tagetes, opium, the lactuca virosa, poppies, and tea when recent, have the power of producing inebriation before they cause stupefaction. Many odours of vegetables, when very powerful, produce also similar effects; as tuberoses, jonquils, lilies, syringa, saffron, fraxinella, &c.

The nauseous narcotics are evident poisons, and are rejected by the taste. They were in old times called *cold poisons*, because they appear to render languid vital action; but they are ordinarily accompanied with a more or less irritating and acrid principle, which produces violent convulsions in the midst of the fatal torpor. Such are the plants of the family of solanums in general, the species datura, hyoscyamus, atropa, nicotiana; several fetid champignons, cicuta, phellandrium, ledum palustre, &c. Almost all the solanums, especially the species having black foliage or fruit, develop, in some part of their structure at least, a narcotic and nauseating principle, as the hyoscyamus, belladonna, and stramonium.

After these generalities, the author enters into the particular consideration of medicinal substances. He first adduces a classification of the qualities he supposes them to possess, than which none can be more objectionable, or show less knowledge of the physiology of the human body. He says, "We may form a graduated scale of the properties of remedies, which will be the inverse of the state of body of the patient. For example, we may range thus the principal class of medicines: 1. Narcotics; 2. *Delayans*, (we give the original, because this is a word of no meaning to English physicians: the truth is, that it has not its equivalent term in the English language, and we shall not furnish matter for ridicule to some English Moliere, by paraphrasing it); 3. *Adoucissans;* 4. Nutritives; 5. Fortifiants; 6. Excitants; 7. Purgatives; 8. Acrids." This is too weak a thing for us to attack: besides this, M. Virey, when speaking of particular medicines, describes them as *incisives, discussives, exsiccatives, depuratives, cephalics;* all which terms, as applied to medicines, are to us as devoid of meaning as the hieroglyphics on the sarcophagus of Alexander.

Mr. Virey distinguishes medicines in the first instance into those of the *organic* and *inorganic* kingdoms; and the former class is subdivided into animal and vegetable. The definition of these, respectively, has certainly defeated the efforts of the best naturalists; we need not, therefore, be surprised that M. Virey has failed to effect it in a satisfactory manner: yet we expected from him something more nearly correct than what he has produced. He designates animals as being cognizable "by a central cavity for nutrition, by their faculty of perception by sensual organs, by the power they have of changing their place at will, by parts of generation which they preserve all their life, which have the sense of touch at least, and five senses at the most."

Now all naturalists know, that there are animals in which neither nerves nor sensual organs have been seen, which cannot change their place at will, which have no generative organs, and which have no more sense of touch than some plants.

M. Virey first treats of medicines taken from the animal kingdom. Almost the sole thing here, after giving the natural history of the animals which supply them, is to state those which *have been*, rather than those which *are now*, in use. The account is a tolerably good one; but the flighty disposition of the author often leads him from his proper object. Thus, who would expect to find the following piece of morality in the first page of this part of the work: "L'HOMME, *homo sapiens, L.*

*(nosce te ipsum).*" And, when speaking of the *cock* and *hen*, that "Les Anglais aiment encore les combats de coqs;" even were this assertion true: and, though he often qualifies his statements of the marvellous virtues of some things, as the secundines of a woman, the dried penis of a stag, the distilled water of a cow's urine, &c. with a *dit-on*, or *on croit*, or *vertus imaginaires*, he is not always so cautious: thus, he formally asserts himself that the *pediculus humanus* "insinué dans l'urètre, fait uriner dans les stranguries."

The natural history of vegetable medicines is better executed; and we approve of the author's arrangement, in taking the method of Jussieu as the basis of their order. We have only to object to the statements made of their medicinal qualities, which are vague, and often devoid of precise meaning to modern physicians. We know not what ideas the physicians of the last and previous century might have attached to the terms *incisive*, *exsiccatives*, and the rest before enumerated, at all conformable with any of the processes of the animal body; but it is certain, that the generality of physicians of the present day do not pretend to understand them.

He next treats of mineral substances, which he divides into combustible and *combusted* substances, and then of "the general substances of nature," which might, he says, "be called elementary." They form two classes: 1st. That of substances coercible by our mechanic powers, as water, air, or gases; 2d. That of incoercible substances, as caloric, light, electricity, magnetism.

A more interesting and more original disquisition follows on *Aliments*, which we shall consider in a particular manner in another article.

